# Anatomical curve identification

**DOI:** 10.1016/j.csda.2014.12.007

**Published:** 2015-06

**Authors:** Adrian W. Bowman, Stanislav Katina, Joanna Smith, Denise Brown

**Affiliations:** a School of Mathematics & Statistics, The University of Glasgow, UK; b Institute of Mathematics and Statistics, Masaryk University, Brno, Czech Republic; c MRC/CSO Social & Public Health Sciences Unit, The University of Glasgow, UK

**Keywords:** Anatomy, Change-point, P-splines, Principal components, Principal curves, Shape analysis, Smoothing

## Abstract

Methods for capturing images in three dimensions are now widely available, with stereo-photogrammetry and laser scanning being two common approaches. In anatomical studies, a number of landmarks are usually identified manually from each of these images and these form the basis of subsequent statistical analysis. However, landmarks express only a very small proportion of the information available from the images. Anatomically defined curves have the advantage of providing a much richer expression of shape. This is explored in the context of identifying the boundary of breasts from an image of the female torso and the boundary of the lips from a facial image. The curves of interest are characterised by ridges or valleys. Key issues in estimation are the ability to navigate across the anatomical surface in three-dimensions, the ability to recognise the relevant boundary and the need to assess the evidence for the presence of the surface feature of interest. The first issue is addressed by the use of principal curves, as an extension of principal components, the second by suitable assessment of curvature and the third by change-point detection. P-spline smoothing is used as an integral part of the methods but adaptations are made to the specific anatomical features of interest. After estimation of the boundary curves, the intermediate surfaces of the anatomical feature of interest can be characterised by surface interpolation. This allows shape variation to be explored using standard methods such as principal components. These tools are applied to a collection of images of women where one breast has been reconstructed after mastectomy and where interest lies in shape differences between the reconstructed and unreconstructed breasts. They are also applied to a collection of lip images where possible differences in shape between males and females are of interest.

## 1 Introduction

Medical imaging has a very wide variety of forms, each of which provides insight into the anatomy of the human body. The technique of stereo-photogrammetry cleverly uses the mismatch between images from two or more adjacent cameras to reconstruct the three-dimensional surface shape of an object of interest. This type of imaging has been used in a wide variety of applications. For example, in surgery there is a strong need to audit the outcome of operations by quantifying the resulting shape in a suitable way. Duffy et al. (2000), Hood et al. (2003) and Nkenke et al. (2006) discuss this in the context of surgical correction of facial shape in children suffering from a cleft lip and/or palate. Wider biological and diagnostic issues associated with facial shape are discussed by Andresen et al. (2000), Hammond et al. (2005) and Hennessy et al. (2010), using laser-scan images which produce data of a very similar structure. Recently, Henseler et al. (2011) have used stereo-photogrammetry to collect images of the female breast in order to quantify the success of surgical reconstruction after mastectomy. The data which these images provide offers a very helpful route to quantitative analysis, as opposed to more subjective assessment of the outcome.

A natural and traditional starting point for the analysis of anatomical shape is to identify a set of landmarks, which are points on an anatomical surface which can be reliably identified. Methods of statistical analysis for landmarks are very well developed, with an excellent description given by Dryden and Mardia (1998). However, the expectation of this approach is that the number of landmarks will be small or modest and this cannot therefore do justice to the very rich surface representations, characterised by thousands of three-dimensional points, produced by stereo-photogrammetry with modern, high-resolution cameras, or by laser scanning.

This paper discusses the identification of anatomical curves, with the aim of providing a much richer characterisation of surface shape than landmarks alone and as a potential intermediate step to a suitable characterisation of the full anatomical surface. In particular, curves often define the boundaries of anatomical features of interest, allowing the position of these to be identified and, if appropriate, extracted from the larger object for separate analysis. There are many examples of the use of curves derived from surfaces in the computer vision and computer graphics literature, such as the construction of crest lines described by Stylianou and Farin (2004) and the detection of ridges and valleys discussed by Lopez et al. (1999). Differential geometry is a standard tool to characterise surface shape in general. The aim of the present paper is to use these ideas in the context of statistical methods which can accommodate the presence of noise in the surface representation and which can weigh up the evidence for the presence and location of features of interest through appropriate statistical tools. The estimation of ridges, valleys and other surface features is a challenging problem, even in the case of more standard response surface settings, as discussed by Hall et al. (2001) for example. However, in anatomical settings the expected shapes of features of interest can be exploited to guide estimation in a very helpful manner.

**Fig. 1:**
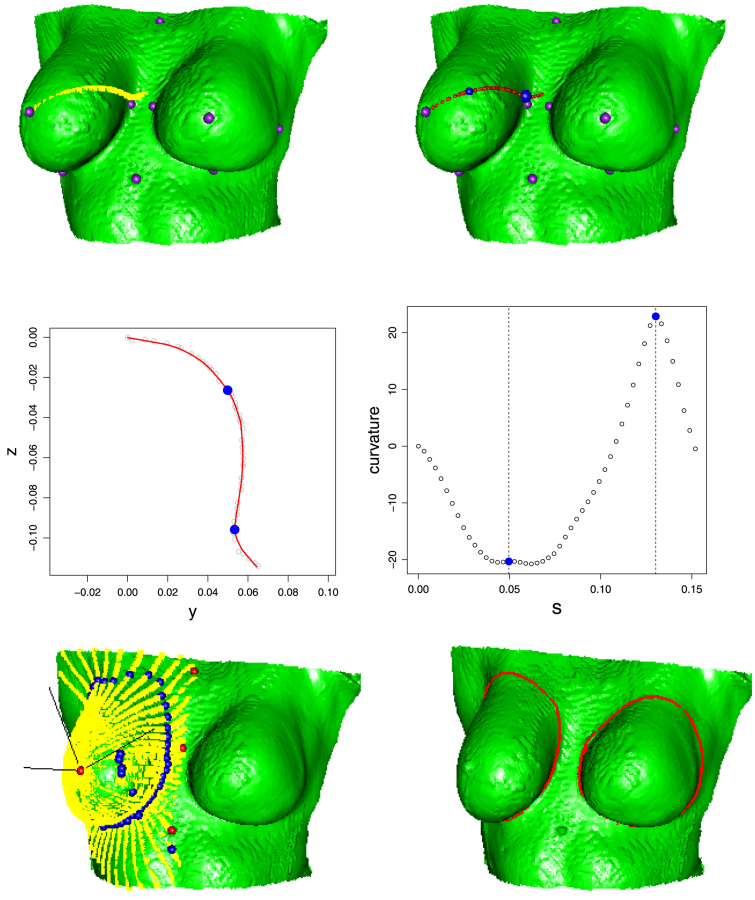
The top row of pictures shows an image of a female torso with a strip of points defined by a planar cut (left) and a principal curve fitted to this strip (right), with points of maximum curvature highlighted in blue. The larger blue point is the one identified as lying on the boundary curve. In both pictures, anatomical landmarks are shown in purple. The middle left hand plot shows the principal curve in detail, with the points of local maximum curvature identified in blue. The middle right hand plot shows curvature as a function of arc length along this principal curve. The lower left hand image shows an axis system located at the most prominent point and associated with the principal curve in the upper plots. A full set of radial strips plus their associated points of maximum curvature is also displayed. The lower right hand panel shows the final boundary estimates derived from principal curves through the points of maximum curvature, for both left and right breasts. (For interpretation of the references to colour in this figure legend, the reader is referred to the web version of this article.)

Two specific applications are considered in the paper. One is to identify the boundary of female breasts on the surrounding chest wall. This is an initial step in comparing the shape of a breast which has been reconstructed after surgery with the shape of the unaffected breast from the same woman. The upper panels of Fig. 1 illustrate one of the 44 raw images which were collected by a stereo-camera system described by Henseler et al. (2011). This data collection system was designed especially for this study and the resulting images are therefore unusual. Initial analysis, reported by Henseler et al. (2012), was based on 10 anatomical landmarks, shown as purple points in the upper panels of Fig. 1. These aim to characterise large scale features such as the most extreme lower and lateral extents of each breast but well defined and reproducible landmarks can be difficult to identify and, in any event, such a small number of point locations clearly does not fully capture the richer anatomical shape expressed in the surface of the image.

A second application focuses on human lips, whose location and shape is of considerable interest in a variety of settings. They form an important part of facial expression, studied in general by Schyns et al. (2009), while the characterisation of normal lip shape provides a reference against which the success of surgical correction can be evaluated, as described by Ayoub et al. (2011) in the case of cleft lip. The lower panels of Fig. 4 illustrate one of the 47 lip images collected as a control sample, as described by McNeil (2012). Thirty-five of the subjects were female and there is interest in possible sexual dimorphism in lip shape. Seven traditional anatomical landmarks are identified on the images.

To avoid laborious manual identification, it would be very convenient to have an automatic method to locate the lips on an image. Most of the approaches discussed in the literature are based on detection of the lip boundaries on two-dimensional images, where the change in colour from lip to skin is the key information. In a standard red–green–blue representation, the red and blue components are present to similar degrees in both lip and skin colour, as reported by Eveno et al. (2001), and so the green component is the most informative, with Hulbert and Poggio (1998) proposing the contrast between red and green as the basis of discrimination. However, as discussed by Kakumanu et al. (2007), there can be substantial difficulties in the use of colour, not least because the camera images are strongly influenced by lighting conditions and shading. In addition, some forms of imaging such as laser scanning may not always provide colour information.

In terms of methods for the detection of boundaries, standard approaches in the computer vision literature include *active contour models* (also known as *snakes*) discussed by Kass et al. (1987) and Delmas et al. (1999) for example, *active shape models* and *active appearance models* discussed by Cootes et al. (1994) and Matthews et al. (2002). Snakes are general models designed for edge and contour detection which can sometimes run into difficulties with features such as lip corners, although these can be identified in the absence of landmarks using methods described by Eveno et al. (2002) and Ozgur et al. (2008). Fully automatic methods are difficult to construct, so snakes were designed for a context where the user can give interactive input to provide visual adjustment and overcome sensitivity to initial settings of the algorithm. Active shape models and active appearance models are constructed around a training set of annotated cases and they also assume a suitable initialisation. These methods have been explored mostly in two-dimensional images, although Krueger et al. (2008) described the use of active contour models in three-dimensional facial settings.

The approach investigated in this paper is motivated by the three-dimensional setting and it is based on geometric characteristics of surface shape. The general strategy for navigating across an anatomical surface, and the particular issues associated with identifying the boundary between a breast and its surrounding chest wall, are discussed in Section  2. The effectiveness of these methods, and their application in characterising shape variation across samples of images, are discussed in Section  3. The same general principles are applied to the identification of curves which define the shape of lips in Section  4, where additional issues of recognising the presence of features characteristic of a lip are addressed. The application to the characterisation of shape variation across samples of images are discussed in Section  5. Some final discussion is given in Section  6.

## 2 Breast boundary identification

Each anatomical surface of interest constitutes a two-dimensional manifold M embedded in three-dimensional space $R^{3}$ and the aim is to identify a breast boundary curve b which lies within M. The issue of surface navigation therefore immediately arises, as it is necessary to remain on the manifold M while constructing an estimate of b. A co-ordinate system which indexes locations within M, but does not index locations outside M, is required. In practice, M is actually characterised by a set of three-dimensional points $\left\{p_{i}:i=1,\ldots ,K\right\}$ for some large K, generally around 30,000 for torso images. The locations of these points are a product of the stereophotogrammetric matching algorithm and they are essentially unstructured, with no points having particular anatomical interpretations.

The approach taken below is to construct local co-ordinate systems through planar transects of the surface, which create one-dimensional planar curves. This reduces the dimensionality of the problem and allows the identification of boundary points through locations of maximum curvature. The boundary curves can then be identified by suitable collation of these identified boundary points. The details of this strategy are discussed below.

### 2.1 Surface navigation

As a starting point, it is relatively easy to locate the most prominent point (*prom*) on the breast, either manually or as the most extreme point in a suitably oriented image. At this location, a set of orthogonal axes in three-dimensional Euclidean space can be constructed, centred on *prom* and with one axis in the direction of the normal to the surface at this location. The normal direction is easily located by considering a principal component analysis of the most prominent point and its immediate neighbours. A suitable definition of a neighbourhood is points lying within a distance of 1.2 cm of *prom*, as this provided around 65 points on average, to ensure stable identification of the principal components. The first two principal component directions, say $n_{1}$ and $n_{2}$, define a plane through these points while the third principal component lies in the normal direction, $n_{3}$. The bottom left hand image of Fig. 1 shows these axes. The plane defined by $n_{2}=0$ then cuts the surface in a perpendicular manner. The curve representing the edge of this cut is characterised by the set of points $\left\{p_{i}:\left|p_{i}^{T}n_{2}\right|\leq \delta ;i=1,\ldots ,K\right\}$, for some small tolerance δ, taken to be 1 mm in view of the high resolution of the surface image. In order to create a radial transect from *prom*, containing points which lie in one particular direction but not those lying in the opposite direction, this also requires that projection onto direction $n_{1}$ is positive, giving the final characterisation of a radial strip as

$$S=\left\{p_{i}:\left|p_{i}^{T}n_{2}\right|\leq \delta ;p_{i}^{T}n_{1}>0,i=1,\ldots ,K\right\}\text{.}$$

The top left panel of Fig. 1 gives an example of points defined in this way, highlighted in yellow.

A proper curve representation is required and a very convenient solution is provided by the concept of a principal curve, introduced by Hastie and Stuetzle (1989) as a flexible form of principal component analysis which generalises the usual linear approach.

A curve lying in a plane can be written as f(s)={x(s),y(s)}, where s indexes the length of the curve from its initial point and x(s),y(s) describe the movement of the x and y co-ordinates as s moves from 0 to its terminating point. The projection of any point p onto a curve can be defined simply as the closest point on the curve, characterised by the corresponding arc length $s_{f}\left(p\right)=\left\{s:\left\|p-f\left(s\right)\right\|=\min_{s}\left\|p-f\left(s\right)\right\|\right\}$. A principal curve is then defined by the special property that its location at any point of interest is the mean value of the points which project there, namely $E_{P}\left\{P|s_{f}\left(P\right)=s\right\}=f\left(s\right)$. When a principal curve is estimated from observed data, this expectation is replaced by a smoothing procedure which estimates the co-ordinates by local averaging. A wide variety of choices are available for the smoothing operation involved, including for example *lowess* (Cleveland, 1979), more general local linear methods (Fan and Gijbels, 1996), smoothing splines (Green and Silverman, 1994) and p-splines (Eilers and Marx, 1996). Broadly speaking, the precise mechanism of smoothing is less important than the choice of amount of smoothing, conveniently expressed in approximate degrees of freedom. Wood (2006) gives a helpful overview of the issues associated with smoothing and of the different approaches available. The idea of a principal curve has found a wide variety of applications, as described for example by Banfield and Raftery (1992). The technical details of construction are described by Hastie and Stuetzle (1989) and well defined algorithms and associated software, such as the princurve package (Hastie and Weingessel, 2011) for R (R Development Core Team, 2013), are widely available.

The middle left hand image of Fig. 1 shows the identified strip points as a two-dimensional pattern, in the plane defined by $n_{2}=0$, with a principal curve superimposed. Notice that there is very little noise in this anatomical setting, because of the high-resolution of the stereo-photogrammetry data, although there is useful suppression of minor imperfections in the captured surface. The curve displayed here is based on a smoothing spline procedure using 6 degrees of freedom. Since we expect the curve to consist of two very smooth pieces, joined at a more rapidly changing junction point where the breast meets the surrounding torso, this modest increase in flexibility over simple linear or quadratic shapes is appealing and the suitability of this choice has been endorsed by repeated effective use over a wide variety of breast curves of this type.

### 2.2 Identification of boundary points

A major benefit of the strategy of fitting principal curves to the data from surface transects is that the complexity of the problem is reduced from the full two-dimensional manifold M to a one-dimensional curve {x(s),y(s)} in a particular radial direction of interest. The breast boundary along this curve can then be located as the point with highest curvature, where the breast joins the chest surface. Curvature is easily computed from the functional parameterisation through the well known formula

$$\kappa \left(s\right)=\left(x^{\prime }y^{\prime \prime }-x^{\prime \prime }y^{\prime }\right)/{\left(x^{\prime 2}+y^{\prime 2}\right)}^{3/2}\text{,}$$

described for example by Koenderink (1990). The derivatives required are easily computed from the spline representation of the curve but are also readily available from other smoothing techniques. Normally, the estimation of derivatives is subject to considerable variability but this is substantially ameliorated in the present context by the high resolution nature of the data. The middle right hand panel of Fig. 1 shows the curvature as a function of s, from which points of local maximum curvature are easily located. (The first of these is a spurious point which, although a local maximum, can easily be excluded because the curvature is negative.) By repeating this process as the axis system shown in the bottom left hand panel of Fig. 1 is rotated clockwise around the axis $n_{3}$, a set of points characterising the breast boundary can be identified. The lower left hand panel of Fig. 1 shows (in blue) the resulting candidate points for the position of the breast boundary.

At this stage, some practical issues arise. One is how far to extend the surface transect, to ensure that the point of maximum curvature is covered, without encroaching on other anatomical features, such as the other breast. A simple solution for directions towards the other breast is to stop the transect at the plane which contains the two landmarks, *xipho* and *ssn*, on the midline of the torso and lying approximately orthogonal to the surface. In other directions, the length of the transect can be constrained to be within a multiple of 1.5 of the maximum distance along the curve of the points of local maximum curvature in the two previous principal curves.

**Fig. 2:**
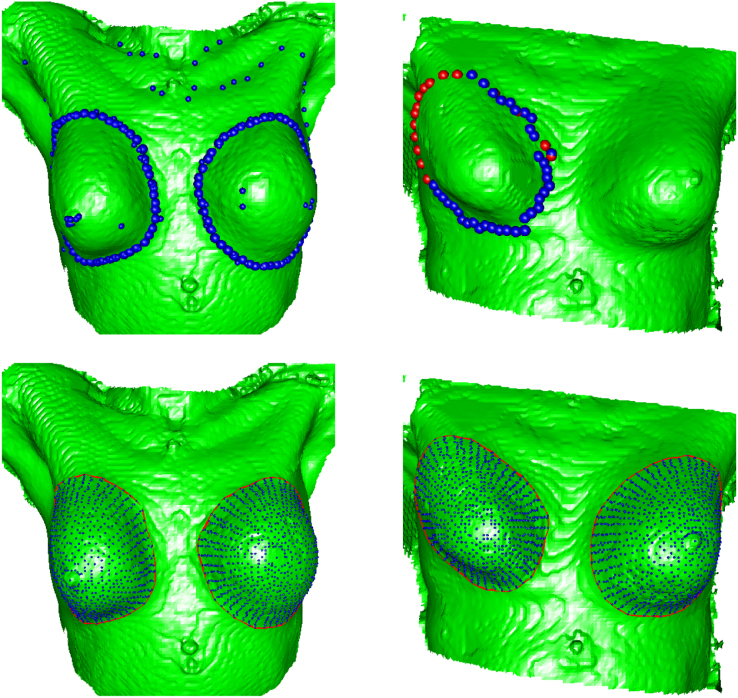
The upper left hand image gives an example where multiple points of maximum curvature are detected along the radial transects, both inside and outside the real breast boundary. The upper right hand image illustrates on a different patient the result of interpolation (red) from single radial points (blue) to areas where more than one radial point was detected. The lower images show a semi-landmark representation of the breasts for both of these cases. (For interpretation of the references to colour in this figure legend, the reader is referred to the web version of this article.)

A more awkward problem is that the stereophotogrammetric reconstruction of the torso surface can create artificial small bumps and pits, which in turn create local maxima in the curvature, some of which may be strong. Genuine anatomical features away from the breast can also generate local curvature maxima. An example of this problem is shown in the upper left panel of Fig. 2. The inappropriateness of these locations as breast boundary points is clearly identified through visual inspection but more automatic implementation of this process is required.

A simple strategy is to make use of the manually identified landmarks (purple points in Fig. 1) as very helpful reference points against which candidate breast boundary points can be compared. For example, boundary points cannot

(a) lie too close to *prom*,
(b) have a protrusion (along the average of the normal vectors at *ssn* and *xipho*) which differs greatly from those of the landmarks at the medial (*med*) and lateral (*lat*) sides of the breast,
(c) have an elevation (along the vector from *xipho* to *ssn*) which differs greatly from those of *ssn* and *inf* (the inferior (lowest) landmark on the breast boundary).

If the average of the normal vectors at *ssn* and *xipho* is denoted by n and the vector from *xipho* to *ssn* by m, then these conditions on a candidate point c are easily expressed in the requirements

(a) ‖c−prom‖>0.7min{‖med−prom‖,‖lat−prom‖},
(b) $\min\left\{med^{T}n,lat^{T}n\right\}-1\;\text{cm}\leq c^{T}n\leq \max\left\{med^{T}n,lat^{T}n\right\}+1\;\text{cm}$,
(c) $inf^{T}m-1\;\text{cm}<c^{T}m<ssn^{T}m$,

where ‖⋅‖ denotes Euclidean distance. The specific constants used here (proportion 0.7 and tolerance 1 cm) are plausible values from subjective judgement but were shown to be effective by experimentation with alternative settings. The success of this strategy is illustrated by the top left hand panel of Fig. 2, where the excluded (smaller blue points) and retained (larger blue points) candidates have been identified correctly.

### 2.3 Identification of boundary curve

A final estimate of the boundary curve b can be constructed by fitting a principal curve $\overset{ˆ}{b}$ to the set of retained candidate points. In order to allow a good degree of flexibility in the boundary shapes which may be present, 12 degrees of freedom were used in the smoothing procedure. Again, this choice was made on the basis of experimentation over a range of different breast shapes.

A further problem is that there may be some radial transects where no candidates for the boundary location are identified. This is most likely to be true in cases where there is a pronounced ridge above the breast as a result of patient pose, as illustrated in the top right hand panel of Fig. 2. The solution adopted for a gap of l radial transects was to extend the principal curves ${\overset{ˆ}{b}}_{L}$ and ${\overset{ˆ}{b}}_{R}$, constructed from the candidate points on the left and right sides of the gap, to produce predicted values ${\overset{ˆ}{b}}_{Li}$ and ${\overset{ˆ}{b}}_{Ri}$ for each radial transect i with a missing estimate of a boundary value. An estimate of the position of the boundary curve in the gap was then provided by a weighted average of these predicted values as

$$\frac{\left(l+1-i\right){\overset{ˆ}{b}}_{Li}+i{\overset{ˆ}{b}}_{Ri}}{l+1}\text{,}\;\text{for }i=1,\ldots ,l\text{.}$$

The top right hand panel of Fig. 2 shows a case where substantial gaps have been interpolated (in red) very successfully in this manner.

Clearly it would be helpful to have a quantitative assessment of the performance of the boundary curve estimation procedure. However, in this setting, there is no natural and convenient means of identifying the ‘true’ boundary curve. Even simple anatomical landmarks are difficult to identify in shapes which are as rounded and unstructured as a breast. Success of estimation therefore has to be assessed by visual means. The estimated boundary curves illustrated in the lower panels of Fig. 2 are typical of the results achieved, indicating a high degree of success. More quantitative assessment of curve estimation is feasible in the case of lip images, as discussed in Section  5.

Breast images are intrinsically complex, including unpredictable effects such as unusual curvature induced by the artificial pose and ‘orange peel’ patterns often created over smooth surfaces by the stereo-photogrammetric reconstruction. These effects make a fully automatic method of boundary detection very difficult to achieve. The algorithm described above has proved very successful, with a visually excellent estimate of the breast boundary immediately identified in a substantial number of cases (more than two thirds). However, it is unrealistic to expect that curves can be accurately identified in a stable and fully automatic manner in every case. This is borne out by Caffo et al. (2008) who used modified versions of principal curves to estimate the three-dimensional centre lines of colon images but who recommended visual inspection and manual intervention as part of the process. The same strategy was adopted here, with adjustment of one or more of the constants in the exclusion criteria at the end of Section  2.2 providing a convenient control mechanism. This makes the procedure semi-automatic, but the improvement over manual curve identification is enormous.

## 3 Analysis of regularised breast images

While the boundary curves identified in the breast data are of intrinsic interest, they are also valuable as a stepping stone in the representation of the entire breast surfaces. These surfaces can be represented as a dense set of discrete points, referred to as *semi-landmarks*, which are appropriately spaced across the surface within the boundary curves. A point-based approach allows standard techniques for the analysis of shape data to be carried out in a straightforward manner since parameter vectors are derived from the coordinates of the fixed landmarks and semi-landmarks. When the dataset is large or landmarks are difficult to place, a functional data form may be preferred, as described by Ramsay and Silverman (2005). Standard shape analysis techniques may still be carried out, as described by Larsen (2005), but at the expense of computational complexity.

**Fig. 3:**
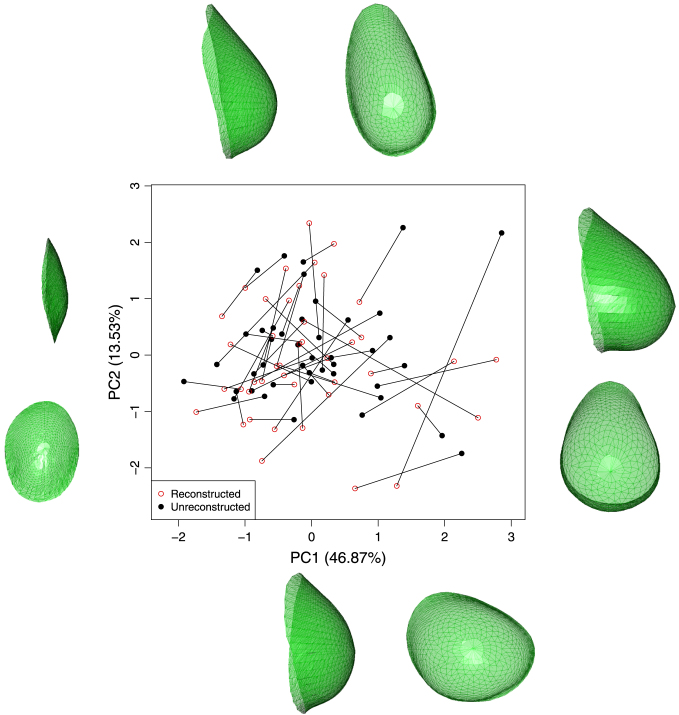
The scores of the first two principal components of the reconstructed (open circles) and unreconstructed (filled circles) breast pairs, joined by lines. The natures of the shape changes associated with these scores are illustrated in the marginal images which correspond to 2 standard deviations in each direction from the mean shape, for principal component 1 (left–right) and 2 (bottom–top).

**Fig. 4:**
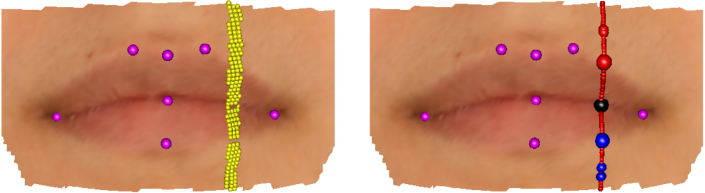
The left hand picture shows a lip image with a strip of points defined by a planar cut. The right hand picture shows a principal curve fitted to this strip, with change-points identified on the lower, midline and upper lip boundaries (blue, black, red respectively). The larger points are those identified as lying on the boundary curves. In both pictures, anatomical landmarks are shown in purple. (For interpretation of the references to colour in this figure legend, the reader is referred to the web version of this article.)

For the breasts, semi-landmarks can be identified at standardised positions along the principal curves fitted to the radial transects. If the number of transects, the starting orientation, the numbers of points on each transect and their proportionate distances along the transect are all held constant then there is a point-to-point correspondence across the breasts within and between individual patients. As all transects converge at the most prominent point, it is beneficial to select points more sparsely towards this end of the strip, in order to obtain a more regular spacing across the surface. To achieve this, a sequence of points $\tau_{i}\;\left(i=1,\ldots ,r\right)$ can be constructed from equally spaced quantiles of the exponential distribution and normalised to fit in the range (0,1), thus ensuring a decreasing distance between points as the strip is traversed. It is then necessary to find the location of each transect at each proportional distance $s_{i}=\tau_{i}s_{\max}$, where $s_{\max}$ denotes the full arc length of the transect of interest. The resulting points are given by $\left(x\left(s_{i}\right),y\left(s_{i}\right),z\left(s_{i}\right)\right)$. By repeating this process across all strips, a representation is obtained of the entire breast surface. For the breast data, r=20 points were found on each of k=51 transects, resulting in a surface representation of 1021 points (including the most prominent) for each breast. These points could then be displayed as a rendered surface by using of the geometry package (Barber et al., 2012) in R to create a triangulation. The marginal images of Fig. 3 illustrate this. This surface representation can now be used as the basis for statistical analysis.

The aim of the study was to assess the differences in shape between the reconstructed and unreconstructed breasts from each woman. Principal components, based on the semi-landmarks, offer a convenient and effective means of describing the main modes of variation in breast shape. Dryden and Mardia (1998) give the details of how this is performed for shape data. Fig. 3 displays the scores of the first two principal components obtained from the breast data, with the associated shape changes displayed in the margins of the plot. The first represents the variability in size of the breast, with larger size accompanied by a fuller and more ptotic (drooping) shape, while the second component captures the rotational and length differences. The reconstructed–unreconstructed pairs are joined by lines. Although large differences are apparent within individual breast pairs, there is no clear indication of systematic differences. This is confirmed by analysis of the differences of the matched scores, where the mean is not significantly different from zero at the 5% level. The analysis of the breast images, of which anatomical curve estimation was a crucial part, has therefore been able to deliver helpful insight into the size and nature of shape variation and the effects of reconstructive surgery.

## 4 Lip boundary identification

The human face is of great interest from social, psychological, medical and biological perspectives and data in the form of three-dimensional images can be collected relatively easily through stereophotogrammetry or laser scanning. Here the former is used, with each facial image represented by around 150,000 points. In this section, the ideas and methods of anatomical curve identification discussed in the earlier sections of the paper are applied to the identification of lip boundary curves. The capture protocol places each subject in a rest position, with closed mouth, and interest lies in evidence of sexual dimorphism, namely whether men and women have systematically different lip shapes.

### 4.1 Surface navigation

**Fig. 5:**
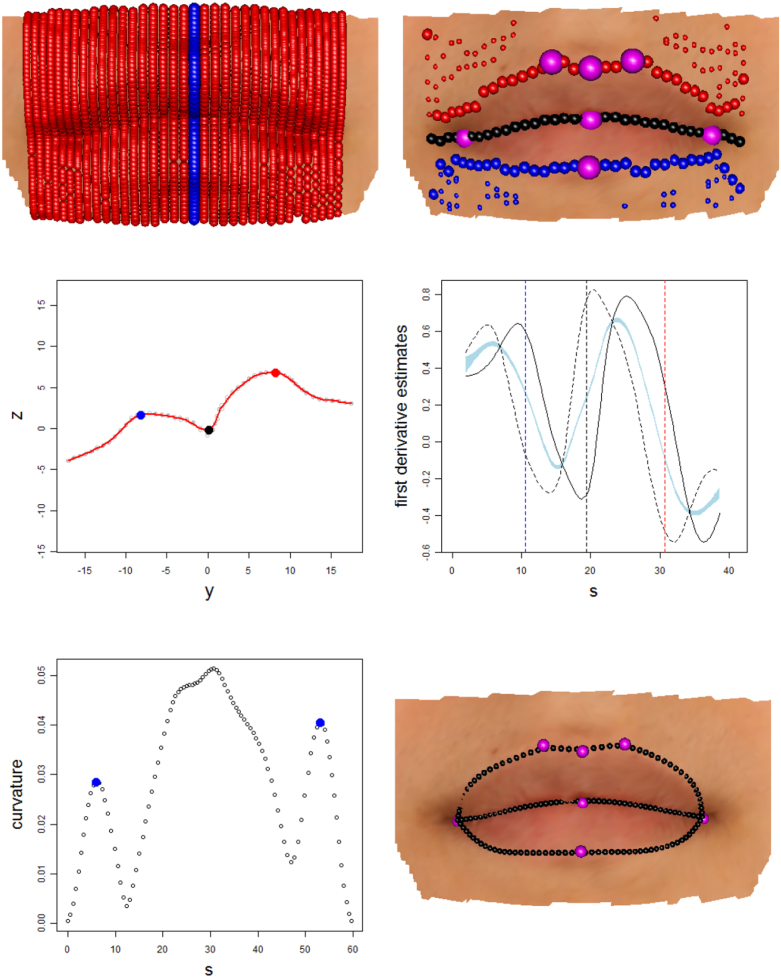
The top left hand panel shows a set of principal curves across the lip region, while the top right hand panel applies the derivative change detection procedure to these principal curves. Candidate points for the upper, mid and lower boundaries are displayed as red, black and blue points respectively. The middle panels illustrate the derivative change detection procedure on the principal curve highlighted in blue in the top left hand panel. The middle right hand panel shows the evidence for sharp changes in derivative as a function of arc length. The middle left hand panel shows the principal curve (red) and associated strip points, with estimated locations for lip boundaries highlighted. The lower left hand panel shows the curvature of the midline curve, as a function of arc length, whose maxima are used to identify the corners of the mouth. The lower right hand panel shows the final boundary curves of the lips, with the upper and lower boundaries constrained to meet the midline curve at the identified corners. (For interpretation of the references to colour in this figure legend, the reader is referred to the web version of this article.)

In contrast to the breast case, there is no natural origin lying within the lips and there are also sharp junctions where the two boundary curves meet at the corners of the mouth. While the general approach of using principal curves to construct a co-ordinate system within the surface manifold M again provides an excellent starting point, the detailed methods need to be adapted to the characteristics of this particular anatomical feature. The top left panel of Fig. 5 shows a set of principal curves superimposed on an image of lips to provide a co-ordinate system on the manifold M. As in the breast images, principal components defined on the mouth surface as a whole provide an initial axis system. Given the elongated shape of a mouth, the first component $\left(n_{1}\right)$ runs in a horizontal direction while the second $\left(n_{2}\right)$ runs vertically, approximately perpendicular to all the lip boundary curves, while the third $\left(n_{3}\right)$ runs in a normal direction. The principal curves were constructed from vertical strips of points defined by $\left\{p_{i}:\left|p_{i}^{T}n_{1}\right|\leq \delta ,i=1,\ldots ,k\right\}$, where k=50 and δ was set to 1.2 mm. Eight degrees of freedom were used for the smoothing procedure to accommodate the two peaks and intervening valley expected in the transect curve. This choice was again supported by experimentation.

### 4.2 Identification of boundary points

In the case of a closed mouth, the lip boundaries are characterised by three well-defined curves. The lower and upper boundaries lie on the ridges formed by the different orientations of lip and skin tissue, although this is usually more marked in the upper boundary than in the lower one. The meeting of the lips is characterised by a sharp change in the opposite direction. The aim is to identify the positions where each principal curve crosses these boundary curves. In contrast to the breast case where a boundary location was sought for every radial direction, some of the curves which form the lip co-ordinate system do not cross the lips at all and so simply identifying points of local extremal curvature is not sufficient. An appropriate way of addressing this is to seek evidence that the direction of the surface changes sharply. There is a very large literature on change-point detection, with Carlstein et al. (1994) providing a helpful starting point and Wyse et al. (2011) giving an example of recent approaches. In the facial setting, the model for each surface strip must be able to track smooth evolution between possible sharp changes and so the approach of Bowman et al. (2006) was adopted. This is based on a comparison of two smooth estimates, one using the data below and the other using the data above the current point of interest. This follows ideas proposed by Hall and Titterington (1992) and Müller (1992) and the general approach has strong connections with the ‘edge-detection’ algorithm of Canny (1986), widely used in computer vision. However, Bowman et al. (2006) also provide inferential methods to assess the presence of a discontinuity.

Most work on change-points is directed towards curve discontinuities but in the facial setting the boundary ridges are identified by discontinuities, or sharp changes, in the first derivative of the curve z(s) which corresponds to the depth component z of the principal curve as it moves up the facial surface. Estimation of a first derivative is straightforward in spline smoothing, with the advantage in the present context that the variance of the errors is low, as discussed in Section  2. Local linear methods can also be used, with the estimate of the first derivative at s defined by the value of β which minimises $\sum_{i}{\left\{z_{i}-\alpha -\beta \left(s-s_{i}\right)\right\}}^{2}$. In both cases, estimates ${\overset{ˆ}{z}}_{a}\left(s\right)$ and ${\overset{ˆ}{z}}_{b}\left(s\right)$, based on data above and below s, can be expressed as $w_{a}z$ and $w_{b}z$ where $w_{a}$ and $w_{b}$ are matrices of weights and z denotes the vector of observed depth values. Fan and Gijbels (1996) give the details for local linear methods. Evidence for sharp change is then expressed in

(1) $$\frac{{\overset{ˆ}{z}}_{a}\left(s\right)-{\overset{ˆ}{z}}_{b}\left(s\right)}{\text{se}\left\{{\overset{ˆ}{z}}_{a}\left(s\right)-{\overset{ˆ}{z}}_{b}\left(s\right)\right\}}\text{,}$$

where the standard error in the denominator is simply ${\left(w_{a}-w_{b}\right)}^{T}\left(w_{a}-w_{b}\right){\overset{ˆ}{\sigma }}^{2}$ and the estimate of error variance ${\overset{ˆ}{\sigma }}^{2}$ can be constructed by local differencing, as described by Gasser et al. (1986). Bowman et al. (2006) go on to derive tests, based on the distribution of quadratic forms, for the presence of one or more discontinuity along the curve, and these methods adapt directly to the case of derivative estimation, with only a change in the weight matrices required. However, the statistic (1) provides a very helpful local expression of evidence, on an interpretable standard error scale, which forms the basis of an effective boundary detection procedure.

The top left hand panel of Fig. 5 shows a set of principal curves across the lip region. The middle left hand panel of Fig. 5 shows the strip of surface points associated with the principal curve highlighted in the top left panel. At this stage the role of the principal curve, also shown in the middle left panel, is simply to construct the arc length values s associated with the observed depth values z. The middle right hand panel shows the estimates of the first derivatives created from below and above each point of interest on the s scale. The amount of smoothing was set to 8 degrees of freedom to accommodate the two peaks and intervening valley expected in the transect curve. The shaded region is a graphical device which is centred on the average of the two estimates and whose width is two standard errors of the difference in the estimates. The locations where the standardised differences are largest are highlighted by vertical lines. These are then the estimates of the locations on the s scale where the lower, midline and upper lip curves lie. These locations are shown on the principal curve in the middle left hand panel of Fig. 5.

In contrast to the breast, where a boundary can be sought in every radial direction, some of the principal curves which form the co-ordinate system across the mouth region do not cross the lips at all. The change-point detection procedure provides a means of addressing this, by weighing the evidence for the presence of a boundary curve. This was applied by identifying a midline curve only in those cases where the absolute value of the standardised difference (1) was greater than 5, indicating strong evidence for the presence of this curve. Where a midline curve is detected, all points of local maximum curvature on the underlying principal curve are identified as potential locations of the upper and lower lip boundaries. The upper right hand panel of Fig. 5 illustrates this process and highlights the presence of a large number of candidate points for the upper and lower lip boundaries as a result of minor imperfections (‘orange peel’ effect) in the stereo-photogrammetric reconstruction of the facial surface.

### 4.3 Identification of boundary curves

The top right hand panel of Fig. 5 shows the results of applying the change-point detection method to all of the original vertical strips, with the points lying on the upper, mid and lower boundaries identified as red, black and blue points respectively. An estimate of the lip boundary curves can now be constructed by fitting a three-dimensional principal curve through the relevant sets of candidate points. The midline curve is sufficiently well-defined that each vertical strip produces only one candidate point. However, it is necessary to deal with the cases where a single vertical strip produces more than one candidate point for an upper or lower lip boundary. A strategy similar to that used with the breasts was adopted. The set of single candidate points was used to construct a preliminary estimate as a three-dimensional principal curve through these locations. In cases with multiple candidates, the location whose distance from the preliminary boundary estimate, measured along the vertical principal curve, was smallest was identified as the relevant candidate point. This augmented set of candidate points was then used to construct a new boundary estimate, again as a three-dimensional principal curve.

Points on the midline curve are very well positioned, while some candidates for the upper and lower lip boundaries are more problematic, erroneously tracking other ridges in the skin shape to the side of the mouth. The particularly good identification of points lying on the midline curve, in the sharp valley created where the two lips meet, provides a solution through identification of the points defining the corners of the mouth. These are characterised by very strong curvature, as the midline curve rises sharply to meet the skin tissue of the cheek. This is illustrated in the lower left hand panel of Fig. 5. These landmarks can therefore be identified by fitting a principal curve, now in three dimensions, to the currently identified lower boundary locations, parameterising this as {x(s),y(s),z(s)} and computing the three-dimensional curvature of this curve as

$$\frac{\sqrt{{\left(x^{\prime \prime }y^{\prime }-y^{\prime \prime }x^{\prime }\right)}^{2}+{\left(x^{\prime \prime }z^{\prime }-z^{\prime \prime }x^{\prime }\right)}^{2}+{\left(y^{\prime \prime }z^{\prime }-z^{\prime \prime }y^{\prime }\right)}^{2}}}{{\left(x^{\prime 2}+y^{\prime 2}+z^{\prime 2}\right)}^{3/2}}\text{;}$$

see Koenderink (1990) for details. The resulting curvature, as a function of arc length s, and the identified corners, characterised as the first and last local maxima, are shown in the bottom left hand panel of Fig. 5. For later reference, the left and right corners are denoted by $\left(x_{L},y_{L},z_{L}\right),\left(x_{R},y_{R},z_{R}\right)$, and the associated arc lengths are denoted by $s_{L}$ and $s_{R}$.

Any points lying beyond these identified corners can now be discarded. In addition, the upper and lower boundaries can now be constrained to meet at these locations. A smoothing procedure lies at the heart of the principal curve construction, as discussed in Section  2, and constraints can be incorporated in a particularly convenient manner with p-spline smoothing. Although the principal curve estimation involves smoothing all three co-ordinates, x,y,z, against arc length s, the process will be illustrated in the case of x. A p-spline curve takes the form of a linear regression, x=Bβ, where the columns of the design matrix B evaluate a set of local, B-spline basis functions at the values of the observed covariate. This is fitted to the observed data by using the regression coefficients $\overset{ˆ}{\beta }$ which minimise the penalised sum-of-squares $S\left(\beta \right)={\left(x-B\beta \right)}^{T}\left(x-B\beta \right)+\lambda \beta^{T}D_{2}^{T}D_{2}\beta$, where the matrix $D_{2}$ creates the second differences of the elements of the β vector. If we wish to force the solution to pass through particular locations, this can be expressed in the constraint Aβ=c, where the columns of the matrix A evaluate the basis functions at the constraint locations and the vector c contains the constrained response values. Specifically, A has two rows which evaluate the basis functions at $s_{L}$ and $s_{R}$, the arc length values at which the left and right hand corner points are located, and c is the vector $\left(x_{L},x_{R}\right)$. The minimisation of S(β) subject to the constraint Aβ=c has a relatively straightforward solution which can be derived by adapting the standard linear model algebra described by Seber (1977, section 3.9). Specifically, the constrained coefficients ${\overset{ˆ}{\beta }}_{c}$ are given by

(2) $${\overset{ˆ}{\beta }}_{c}=\overset{ˆ}{\beta }+{\left(B^{T}B+D_{2}^{T}D_{2}\right)}^{-1}A^{T}{\left[A{\left(B^{T}B+D_{2}^{T}D_{2}\right)}^{-1}A^{T}\right]}^{-1}\left(c-A\overset{ˆ}{\beta }\right)\text{.}$$

It was observed above that estimation of upper lip boundary points can be problematic near the mouth corners. It is therefore extremely helpful that the corner point itself can be very well estimated from the midline curve and the upper boundary curve tied to this location. In addition, the knowledge that a lip curve in this region should not exhibit strong fluctuations can also be used to very good effect by constraining the shapes of the permitted curves. Bollaerts et al. (2006) show how a variety of shape constraints can be induced in p-spline estimates through the use of further penalty terms. Two further sets of penalties were therefore adopted. The first requires the upper lip boundary to be monotonically increasing over the first 40% of its arc length and monotonically decreasing over the final 40%. A similar penalty for the lower lip imposes a decreasing shape for the first 40% and an increasing shape for the final 40%. The second set of penalties require the second derivative of the upper lip curve to be decreasing over the first 40% and increasing over the final 40%. A similar penalty for the lower lip imposes an increasing second derivative for the first 40% and a decreasing second derivative for the final 40%. Since the properties of the curves are inherited from the properties of the coefficients of the B-splines, these conditions can be expressed in terms of first and second differences in the elements of β. The penalty for monotonicity is $\kappa \beta^{T}D_{1}^{T}V_{1}D_{1}\beta$, where the matrix $D_{1}$ constructs the first differences of the elements of β and the matrix $V_{1}$ is diagonal with elements which are 1 when the required monotonicity constraint is violated and 0 otherwise. The penalty for the second derivatives is $\kappa \beta^{T}D_{2}^{T}V_{2}D_{2}\beta$, where the matrix $V_{2}$ is diagonal with elements which are 1 when the change in the second differences of the elements of β has a sign which is inconsistent with the increasing/decreasing criterion for the second derivative described above.

The penalised sum-of-squares function is now

$$S\left(\beta \right)={\left(x-B\beta \right)}^{T}\left(x-B\beta \right)+\lambda \beta^{T}D_{2}^{T}D_{2}\beta +\kappa \beta^{T}D_{1}^{T}V_{1}D_{1}\beta +\kappa \beta^{T}D_{2}^{T}V_{2}D_{2}\beta \text{.}$$

This does not guarantee that the monotonicity and second derivative constraints will be met exactly, but the use of a large penalty parameter κ will induce this behaviour in large measure. These shape constraints themselves force a strong degree of smoothness on the estimated curves and so it is feasible to use a large number of degrees of freedom to specify λ. Here, 25 degrees of freedom were used, particularly to allow good estimation in the central area of the upper lip, where a ‘notch’ is often present. The second penalty parameter κ was then simply set to 100λ. The final result is displayed in the bottom right hand panel of Fig. 5.

With lips, there is an opportunity to quantify the performance of the curve identification procedure through the well-defined and commonly used anatomical landmarks which can be placed manually on a lip image. The lower right hand panel of Fig. 5 shows 7 such anatomical landmarks, all of which should lie on the lip boundary curves. The success of lip boundary estimation can then be quantified by observing how close these manual landmarks are to the estimated curves. For the mouth corners, the mean distance between the manual and automatic points was 1.17 mm (sd 0.58 mm) which represents an excellent level of agreement. This is even better across the five interior lip landmarks, where the mean separation distance was 0.59 mm (sd 0.52 mm). A more general assessment for the curve as a whole is obtained by comparing the estimates from curves which have been manually marked by a trained observer (sk). Table 1 displays the square roots of the average distances between the estimated and marked curves across 51 equally spaced points along each curve. These mean distances are also separated into horizontal (x), vertical (y) and depth (z) directions after orienting each face into a frontal pose. This confirms excellent performance of the curve estimation method, with all average distances below 1 mm and substantially so in many cases.

## 5 Analysis of regularised lip images

For the lips, semi-landmark representations can easily be constructed from surface transects and principal curves between regularly spaced points on the upper (and lower) boundary and the midline. Fifty points were used on each boundary curve. For each lip, the boundary points were taken in pairs and the intervening principal curves across the lip surface were used to create numbers of semi-landmarks which varied according to lip width, with 3 near the mouth corners, rising to 24 at the widest point. This created 896 semi-landmarks for each lip.

As a result of the regularised structure imposed by semi-landmarks, with numbers and positions which correspond across different images, variation in surface shape can again be explored using standard tools such as principal component analysis. A graphical representation of the shape change associated with a particular component can be constructed by identifying the shape associated with a particular score.

**Fig. 6:**
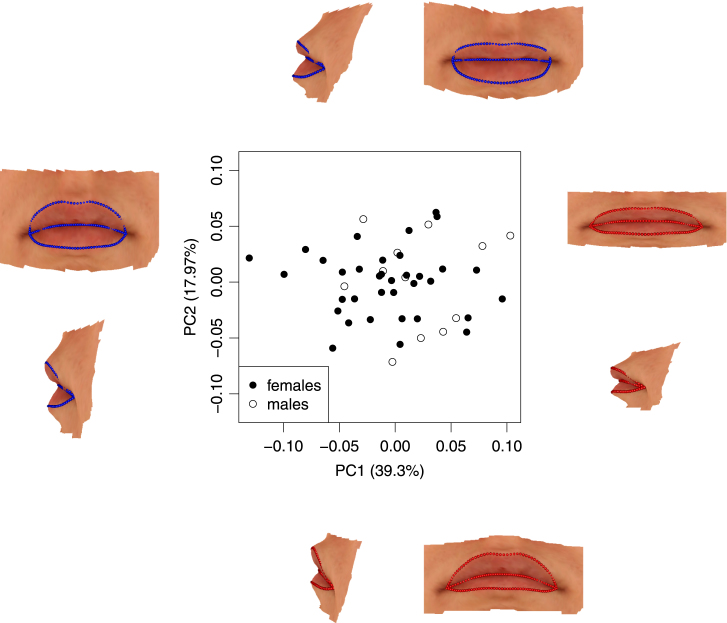
The scores of the first two principal components of the lip shapes for males (open circles) and females (filled circles). The natures of the shape changes associated with these scores are illustrated in the marginal images which correspond to 2 standard deviations in each direction from the mean shape, for principal component 1 (left–right) and 2 (bottom–top).

Fig. 6 displays the first two principal components obtained from the lip data. In this application the surface colour is also a natural component of the image. A thin-plate spline model (Bookstein, 1989) has therefore been applied to warp a particular template surface patch around the mouth to the required principal component score position. This displays the lips in their natural spatial context for visual effect, including appropriate skin colour. The shape changes associated with the first and second principal components are displayed in the margins of Fig. 6. The first principal component (left and right hand images) corresponds to fuller or narrower lips, while the second principal component (top and bottom images) is dominated by asymmetry in the sense that one lip is fuller or narrower than the other. There is a considerable overlap in principal component scores for males and females. There is a slight indication that females are more common at the ‘fuller’ end of the scale for principal component 1, but more formal comparison of the mean scores shows the evidence not to be significant at the 5% level, with a dataset of this modest size.

## 6 Discussion

This paper has been concerned with the identification of curves lying on manifolds, in order to identify the key structures of anatomical surface images in three dimensions. Several significant issues were raised by this problem. The first was navigation across a manifold, which was solved by the use of principal curves. The second was the identification of the boundaries of interest through local geometry. This was approached by a reduction of dimensionality through a local co-ordinate system appropriate to the feature, plus the use of curvature to define the locations of interest. The boundary curves were then constructed by smoothing across the manifold using a further principal curve.

Selecting degrees of smoothing is always an important issue and in general this can be a difficult choice because the form of the underlying function is unknown. However, in the anatomical context choices can be helpfully informed by experimentation, as this is a setting where common characteristics are expected. Similarly, several constants need to be selected, for example to specify distances determining exclusion criteria for spurious locations. However, knowledge of the appropriate anatomy has been built into the algorithm, to take advantage of the key features such as closed curves for breast boundaries, and junction points, monotonicity and curvature constraints in lip curves. The choice of appropriate smoothing and threshold parameters has also been guided by the anatomical context, as well as by experimentation.

In both applications, the boundary curves have been estimated without the use of any manually identified landmarks, except to provide an initial orientation which need not be highly accurate. Where high quality landmarks are available, it would be straightforward to incorporate these into the curve estimation procedures. For example, the adjustment defined by (2) could be used to require the boundary curve to pass through relevant lip landmarks. Conversely, where landmarks are of interest but not available, boundary curves can provide a means of estimating these. This has already been discussed in the case of lip corners but other landmarks, such as the notches in the middle of the upper lip curve, could similarly be identified through points of extreme local curvature.

Curves identified by any algorithmic means, however effective in general, should always be subject to quality control in the form of visual inspection. This remains true with the methods described here, despite the good performance when calibrated against detailed manual identification of landmarks. The possibility of unusual and unexpected features causing significant misalignment of the estimated curves is small but cannot be eliminated completely. This echoes the perspective of Caffo et al. (2008) in a similar application context. However, the availability of an automatic tool which has a high degree of reliability is an invaluable resource which can save a large amount of time which would otherwise need to be spent in laborious manual marking. Even in the occasional cases which are problematic, the estimated curves provide a helpful starting point for manual adjustment.

## Figures and Tables

**Table 1:** The square roots of the average squared distances between estimated and manually marked lip curves, separated into horizontal (x), vertical (y) and depth (z) directions.

	x	y	z	Overall
Lower lip	0.644	0.640	0.467	0.685
Mid lip	0.948	0.349	0.517	0.733
Upper lip	0.658	0.705	0.567	0.741
